# Impaired Antibody Response Following the Second Dose of the BNT162b2 Vaccine in Patients With Myeloproliferative Neoplasms Receiving Ruxolitinib

**DOI:** 10.3389/fmed.2022.826537

**Published:** 2022-03-25

**Authors:** Daisuke Ikeda, Toshiki Terao, Daisuke Miura, Kentaro Narita, Ami Fukumoto, Ayumi Kuzume, Yuya Kamura, Rikako Tabata, Takafumi Tsushima, Masami Takeuchi, Takaaki Hosoki, Kosei Matsue

**Affiliations:** ^1^Division of Hematology/Oncology, Kameda Medical Center, Chiba, Japan; ^2^Department of Hematology, Kimitsu Central Hospital, Chiba, Japan

**Keywords:** COVID-19, antibody response, vaccine, MPN, ruxolitinib, BNT162b2

## Abstract

Data on the effect of ruxolitinib on antibody response to severe acute respiratory coronavirus 2 (SARS-CoV-2) vaccination in patients with myeloproliferative neoplasms (MPN) is lacking. We prospectively evaluated anti-spike-receptor binding domain antibody (anti-S Ab) levels after the second dose of the BNT162b2 (Pfizer-BioNTech) vaccine in MPN patients. A total of 74 patients with MPN and 81 healthy controls who were vaccinated were enrolled in the study. Of the MPN patients, 27% received ruxolitinib at the time of vaccination. Notably, MPN patients receiving ruxolitinib had a 30-fold lower median anti-S Ab level than those not receiving ruxolitinib (*p* < 0.001). Further, the anti-S Ab levels in MPN patients not receiving ruxolitinib were significantly lower than those in healthy controls (*p* < 0.001). Regarding a clinical protective titre that has been shown to correlate with preventing symptomatic infection, only 10% of the MPN patients receiving ruxolitinib had the protective value. Univariate analysis revealed that ruxolitinib, myelofibrosis, and longer time from diagnosis to vaccination had a significantly negative impact on achieving the protective value (*p* = 0.001, 0.021, and 0.019, respectively). In subgroup analysis, lower numbers of CD3^+^ and CD4^+^ lymphocytes were significantly correlated with a lower probability of obtaining the protective value (*p* = 0.011 and 0.001, respectively). In conclusion, our results highlight ruxolitinib-induced impaired vaccine response and the necessity of booster immunisation in MPN patients. Moreover, T-cell mediated immunity may have an important role in the SARS-CoV-2 vaccine response in patients with MPN, though further studies are warranted.

## Introduction

During the coronavirus disease 2019 (COVID-19) pandemic caused by severe acute respiratory coronavirus 2 (SARS-CoV-2), high mortality rates were reported in COVID-19 patients with myeloproliferative neoplasms (MPN). A previous report showed that 28.6% of the MPN patients who developed COVID-19 died, of which those with myelofibrosis (MF) had a higher mortality rate reaching 48% (1). Hence, prevention is of utmost importance, and substantial efforts are being made to expand vaccination against SARS-CoV-2. However, there is a growing concern that vaccination is less effective in haematological malignancies than in healthy individuals due to treatment-induced immunosuppression and disease-related immune dysregulation (2, 3).

Ruxolitinib, a potent and selective Janus kinase (JAK)1/JAK2 inhibitor widely used for MF, suppresses proinflammatory cytokines (4). It also targets various cellular components of the immune system, such as dendritic cells, natural killer cells, and CD4^+^ T-cells, which affect innate and adaptive immunity (4–6). Consequently, this immunosuppressive activity increases the susceptibility to infections in MPN patients (7). On the other hand, its immunomodulatory properties may be beneficial in the inflammatory phase of COVID-19 (8). However, data on whether and how ruxolitinib affects the magnitude of antibody response to SARS-CoV-2 vaccination is lacking (9–13). Here, we report a highly impaired serological response to the second dose of SARS-CoV-2 vaccination in MPN patients receiving ruxolitinib.

## Methods

We prospectively analysed the data of 74 consecutive patients at Kameda Medical Centre and Kimitsu Central Hospital diagnosed with Philadelphia chromosome-negative MPN according to the 2016 World Health Organisation criteria, including polycythaemia vera, essential thrombocythaemia, MF, and unclassifiable MPN. They received their second dose of the BNT162b2 (Pfizer-BioNTech) vaccine between June and October 2021. The vaccine was administered twice at 3-week intervals according to the standard protocol. One patient with polycythaemia vera and transformed acute myeloid leukaemia was excluded because of venetoclax use. For reference purposes, we enrolled age-matched 81 healthy controls (HCs) vaccinated simultaneously and with the same modalities. After the second dose, all participants were assessed for anti-nucleocapsid antibody and anti-spike-receptor binding domain antibody (anti-S Ab) levels. Serum specimens were analysed using the Elecsys® Anti-SARS-CoV-2S assay (Elecsys Anti-SARS-CoV-2 N ECLIA, Roche Diagnostics, Burgess Hill, UK) performed on a Cobas 8000 e801 (Roche Diagnostics). According to the manufacturer's recommendations, anti-S Ab concentrations <0.80 U/mL and ≥0.80 U/mL were considered negative and positive, respectively (14). All statistical analyses were conducted using R version 4.1.1 (The R Foundation for Statistical Computing, Vienna, Austria) and using the EZR software package (Saitama Medical Centre, Jichi Medical University, Shimotsuke, Japan), which is a graphical user interface for R. The Mann–Whitney *U*-test or the Kruskal–Wallis test were used to compare differences between continuous variables. In contrast, Fisher's exact test was used for categorical variables. Univariate analysis was performed using logistic regression. A value of two-sided *p* < 0.05 was considered statistically significant. This study was conducted in accordance with the Declaration of Helsinki and was approved by the ethical review board of each institution. Written informed consent was obtained from all participants.

## Results

Patient and HCs characteristics are summarised in Table 1. There were 32 (43%), 20 (27%), 19 (26%), and 3 (4%) MPN patients with essential thrombocythaemia, polycythaemia vera, MF, and unclassifiable MPN, respectively. The median age was 72.5 years (range, 41–92 years) in MPN patients and 74 years (range, 55–92 years) in HCs. Molecular analysis showed *JAK2* V617F mutation in 47 (63%) patients, *CALR* mutation in 11 patients (15%), *MPL* mutation in 2 patients (3%), and triple-negative mutation in 13 patients (17%). At the time of vaccination, 20 (27%) patients received ruxolitinib (MPN-Ruxo). Of those not taking ruxolitinib (MPN-no Ruxo), 34 (46%) received cytoreductive therapy, including hydroxycarbamide or anagrelide, and 20 (27%) were on supportive care. The median dose and duration of ruxolitinib intake were 20 mg daily (range, 5–40 mg) and 2.1 years (range, 0.2–5.3 years), respectively.

**Table 1:** Patient characteristics.

	**MPN-Ruxo**	**MPN-no Ruxo**	***p***	**HCs**
	**(*n* = 20)**	**(*n* = 54)**		**(*n* = 81)**
Age, median (range)	71.5 (41–85)	73 (44–92)	0.669	74 (55–92)
Sex, *n*, male (%)	14 (70.0)	25 (46.2)	0.06	34 (42.0)
**Diagnosis**, ***n*** **(%)**				–
ET	3 (15.0)	29 (53.7)	NA	
PV	3 (15.0)	17 (31.5)	NA	
MF	13 (65.0)	6 (11.1)	<0.001	
Primary MF/secondary MF	6 (30.0)/7 (35.0)	4 (7.4)/2 (3.7)		
MPN-U	1 (5.0)	2 (3.7)	NA	
Time from diagnosis, years,	6.7 (4.1–11.6)	5.8 (2.8–9.4)	0.642	–
median (IQR)				
**Driver mutation**, ***n*** **(%)**				–
*JAK2*	11 (55.0)	36 (66.7)	0.41	
*CALR*	3 (15.0)	8 (14.8)	NA	
*MPL*	2 (10.0)	0 (0)	NA	
Triple-negative	3 (15.0)	10 (18.5)		
NA	1 (5.0)	–		
WBC (×10^3^/μL), median (IQR)	6.6 (3.6–9.7)	6.3 (3.8–8.6)	0.039	NA
Lymphocyte (×10^3^/μL), median (IQR)	1.1 (0.9–1.6)	1.4 (1.1–1.9)	0.039	NA
**Treatment**, ***n*** **(%)**			NA	–
Ruxolitinib	20 (100)	–		
Cytoreductive therapy	–	34 (63.0)		
No treatment	–	20 (37.0)		
**Time of exposition to**			NA	–
**ruxolitinib**, ***n*** **(%)**				
<1 year	11 (55.0)	–		
≥1 year	9 (45.0)	–		
**Dose of ruxolitinib**, ***n*** **(%)**			NA	–
≤20 mg	13 (65.0)	–		
>20 mg	7 (35.0)	–		
Interval from 2nd vaccine to	42.5 (22.5–74.5)	41.5 (27–64.75)	0.642	41 (29–55)
Ab analysis, median (IQR)				
Anti-S Ab level, median (IQR)	11.35	319.5	<0.001	677
	(2.06–68.17)	(170.25–689.0)		(362–1,191)
**Seroconversion**, ***n*** **(%)**	16 (80.0)	52 (96.3)	0.036	81 (100)
**Achieving protective value**, ***n*** **(%)**	2 (10.0)	31 (57.4)	<0.001	71 (87.6)
**Anti-N antibody positivity**, ***n*** **(%)**	0 (0)	0 (0)	1	0 (0)

*MPN-Ruxo, myeloproliferative neoplasms with ruxolitinib; MPN-no Ruxo, myeloproliferative neoplasms without ruxolitinib; HCs, healthy controls; ET, essential thrombocythaemia; PV, polycythaemia vera; MF, myelofibrosis; MPN-U, myeloproliferative neoplasms, unclassifiable; JAK2, Janus kinase 2; CALR, calreticulin; MPL, thrombopoietin receptor; WBC, white blood cells; IQR, interquartile range; Anti-S Ab, anti-spike-receptor binding domain antibody; Anti-N IgG, anti-nucleocapsid protein IgG, NA: not assessed*.

**Figure 1:**
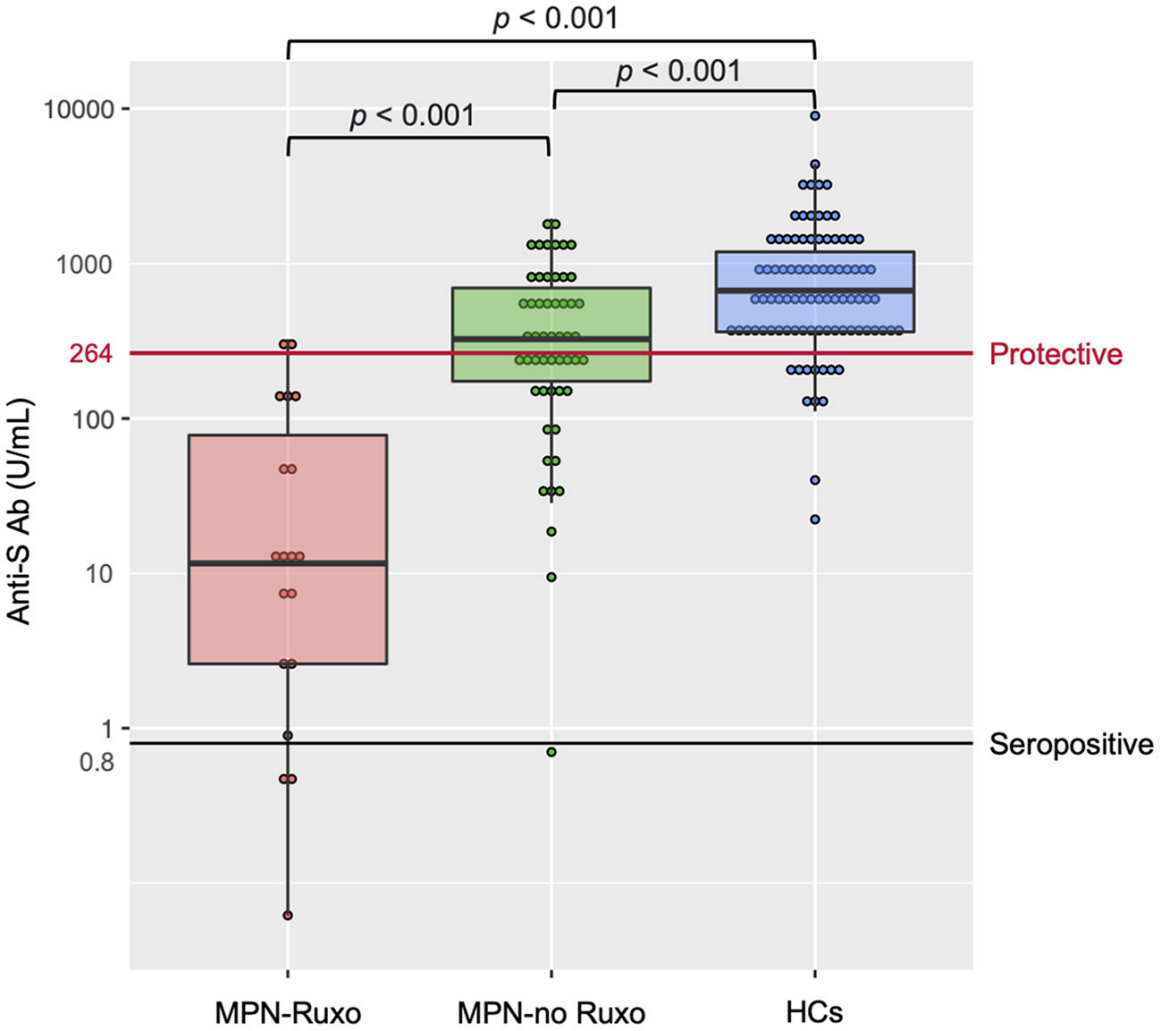
Antibody response after two doses of SARS-CoV-2 vaccine in MPN patients and HCs. MPN, myeloproliferative neoplasms; Anti-S Ab, anti-spike-receptor binding domain antibody; MPN-Ruxo, MPN with ruxolitinib; MPN-no Ruxo, MPN without ruxolitinib; HCs, healthy controls; SARS-CoV-2, severe acute respiratory coronavirus 2.

No patient or HC had detectable anti-nucleocapsid antibody, ensuring no prior SARS-CoV-2 infection. Anti-S Ab levels after the second vaccine dose are shown in Figure 1. The interval from the second vaccine dose to blood sampling was not significantly different between MPN patients and HCs {median 41.5 days [interquartile range (IQR) 27–64.75 days] vs. 41 days [IQR 29–55 days]; *p* = 0.505}. Intriguingly, the MPN-Ruxo group had a highly impaired anti-S Ab response (median 11.35 U/mL [IQR 2.06–68.17 U/mL]) compared with the MPN-no Ruxo group (319.5 U/mL [IQR 170.25–689.0 U/mL]) and HCs (677 U/mL [IQR 362–1,191 U/mL]) (*p* < 0.001 each other). Seroconversion was achieved in 80% of the MPN-Ruxo patients, albeit with low anti-S Ab, 96.7% of the MPN-no Ruxo patients, and 100% HCs. However, breakthrough infection reportedly correlates with the titres of neutralising antibodies linked to anti-S Ab (15), suggesting that being seropositive may not be an indicator of protection against SARS-CoV-2. A recent report showed that 80% vaccine efficacy against symptomatic infection was achieved with anti-S Ab levels of at least 264 binding antibody units (BAU)/mL (16), which was converted by multiplying our antibody concentration by 1 (17). Thus, we established a surrogate endpoint of anti-S Ab ≥ 264 BAU/mL as a protective value and performed further analysis. Only 10% of the MPN-Ruxo patients achieved the protective value compared to 57.4% of the patients in the MPN-no Ruxo group and 87.6% in HCs. Univariate analysis showed that ruxolitinib, MF, and longer time from diagnosis to vaccination (>6 years) were associated with a lower likelihood of achieving the protective value (*p* = 0.001, 0.021, and 0.019, respectively, Table 2). Ruxolitinib use and MF were significantly correlated (*p* < 0.001), although no differences were observed between the time from diagnosis and the former two factors (*p* = 1 and 0.429, respectively). Regarding the manner of ruxolitinib exposure, neither the current dose nor the duration was correlated with reaching the protective value.

**Table 2:** Univariate analysis for obtaining protective levels of antibody to COVID-19 infection after two doses of vaccination.

	**Univariate analysis**		
	**Odds ratio**	**95% CI**	***p***
Age > 70 (years)	0.55	0.21–1.44	0.227
MF	0.23	0.07–0.81	0.021
*JAK2* mutation	0.94	0.36–2.47	0.904
WBC <6,000 (/μL)	0.51	0.18–1.49	0.223
Lymphocyte <1,000 (/μL)	0.41	0.11–1.47	0.172
Time from diagnosis to vaccination > 6 (years)	0.32	0.12–0.83	0.019
Time from 2nd vaccine to Ab analysis > 40 (days)	0.72	0.28–1.81	0.483
Cytoreductive therapy	1.69	0.67–4.27	0.264
Ruxolitinib	0.08	0.01–0.39	0.001

*MF, myelofibrosis; JAK2, Janus kinase 2; WBC, white blood cell; Ab, antibody; CI, confidence interval*.

**Figure 2:**
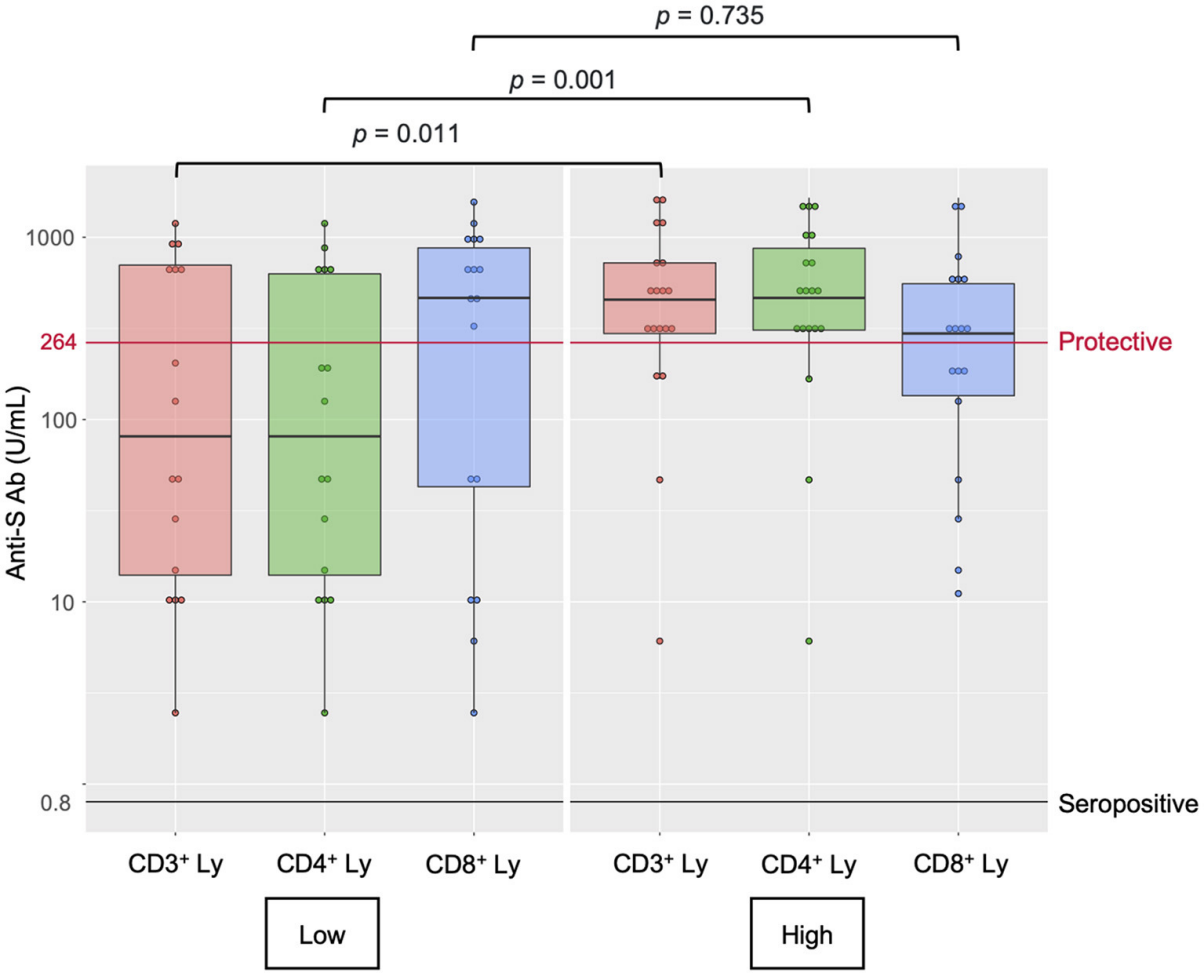
Lymphocyte subset counts and antibody response after two doses of SARS-CoV-2 vaccine in MPN patients. Anti-S Ab, anti-spike-receptor binding domain antibody, Ly, lymphocytes.

Finally, we performed a *post-hoc* analysis of the lymphocyte populations in 36 MPN patients (MPN- Ruxo = 10, MPN-no Ruxo = 26) to explore the immunological profile involving vaccine response (Supplementary Tables 1, 2). The lymphocyte populations were analysed by flow cytometry in samples of peripheral blood mononuclear cells. The MPN-Ruxo group had significantly fewer total lymphocytes, CD3^+^ cells, CD4^+^ cells, and CD56^+^ cells than the MPN-no Ruxo group (*p* = 0.003, 0.007, 0.007, and 0.002, respectively); however, CD19^+^ cells and IgG, which reflected humoral immunity, were maintained. In univariate analysis using the median value as cut-off, low CD3^+^, and CD4^+^ cell counts were significantly associated with a lower probability of obtaining the protective value (*p* = 0.011 and 0.001, respectively, Figure 2). Even in the MPN-no Ruxo group, there was a non-significant correlation between CD4^+^ cell count and protective level of anti-S Ab acquisition (*p* = 0.075).

## Discussion

This study demonstrated a highly impaired antibody response after the second dose of SARS-CoV-2 vaccination in ruxolitinib-treated MPN patients with 30-fold lower median anti-S Ab levels than in those without ruxolitinib. Our result showing the decreased anti-S Ab levels in MPN patients was consistent with previous reports, although the degree of reduction varies (2, 3). On the other hand, the seroconversion rate in MPN patients with ruxolitinib was 80%, which was higher than in previous reports (42–68%) (2, 11–13). Fiorino et al. (13) showed a slower antibody response after second dose SARS-CoV-2 vaccination in MF patients than healthy individuals, regardless of ruxolitinib use. Moreover, Alimam et al. (18) showed delayed and impaired B- and T-memory cell responses to flu vaccination in MPN patients. Taken together, the later timing of antibody measurements in this study (median 41 days after second vaccination) than previous reports may account for the higher rate of seroconversion. However, it remains uncertain whether seropositivity itself provides adequate protection against SARS-CoV-2 in MPN patients who are potentially susceptible to infection (7). Recently, the association of neutralisation titre with protection against SARS-CoV-2 infection has been reported (19). Furthermore, several reports have shown that anti-S Ab is correlated with neutralising antibodies and also with protection against symptomatic and breakthrough infection (15, 16). For example, Feng et al. (16) reported that the anti-S Ab level ≥ 264 BAU/mL 28 days after the second vaccine dose conferred 80% protection against symptomatic infection. Anti-S Ab can be more easily applied in clinical practise with commercially available diagnostic assays than neutralising antibodies, and standardised by converting it into BAU determined by World Health Organisation International Standard (17). Thus, we set anti-S Ab ≥ 264 BAU/mL as the protective value against symptomatic infection. It is noteworthy that only 10% of ruxolitinib-treated MPN patients achieved the protective value. Even without receiving ruxolitinib, the antibody response in many MPN patients was inadequate, with only ~50% of the patients holding protective anti-S Ab levels. These results highlight the necessity of additional immunisation for MPN patients, especially those receiving ruxolitinib.

The mechanism underlying the impaired vaccine response driven by ruxolitinib remains unclear. Time- and dose-dependent negative impacts of ruxolitinib were not observed in our study. Interestingly, the lymphocyte subset analysis showed a negative correlation with low CD4^+^ T-cell counts and a probability of achieving the protective value, particularly in ruxolitinib-treated MPN patients. To the best of our knowledge, this study was the first to describe the lymphocyte population analysis after the second dose of SARS-CoV-2 vaccine in patients with MPN. Sahin et al. (20) demonstrated virus-specific T-cell activation occurred after SARS-CoV-2 vaccination. Furthermore, they showed a positive correlation between CD4^+^ T-cell responses and neutralising antibody titres (20). Regarding the unique immune system changes in patients with MPN, various types of dysregulations, such as T-cell exhaustion, alterations in regulatory T-cells, and natural killer cells dysfunction were reported even in the absence of treatment (4, 21). Therefore, one possible speculation is that ruxolitinib aggravates the T-cell dysfunction inherent in MPN, which responsible for the diminished vaccine response after SARS-CoV-2 vaccination. Our data is limited by the small sample size, the short follow-up period after vaccination, and the heterogenous interval between vaccine administration and blood sampling. Further studies including more patients and longitudinal observation are warranted to confirm these findings.

## Data Availability Statement

The raw data supporting the conclusions of this article will be made available by the authors, without undue reservation.

## Ethics Statement

The studies involving human participants were reviewed and approved by Kameda Medical Centre Institutional Review Board. The patients/participants provided their written informed consent to participate in this study.

## Author Contributions

DI and KM conceptualised and designed the study and wrote the manuscript. DI, TTe, DM, KN, AF, AK, YK, RT, TTs, MT, TH, and KM provided the patient care and collected the data. DI performed statistical analysis. KM supervised the study. All authors critically revised the manuscript.

## Conflict of Interest

The authors declare that the research was conducted in the absence of any commercial or financial relationships that could be construed as a potential conflict of interest.

## Publisher's Note

All claims expressed in this article are solely those of the authors and do not necessarily represent those of their affiliated organizations, or those of the publisher, the editors and the reviewers. Any product that may be evaluated in this article, or claim that may be made by its manufacturer, is not guaranteed or endorsed by the publisher.

## Abbreviations

SARS-CoV-2: Severe acute respiratory coronavirus 2
JAK: Janus kinase
MPN: Myeloproliferative neoplasms
anti-S Ab: Anti-spike-receptor binding domain antibody
COVID-19: Coronavirus disease 2019
MF: Myelofibrosis
HCs: Healthy controls
MPN-Ruxo: MPN patients who received ruxolitinib
MPN-no Ruxo: MPN patients who did not receive ruxolitinib
IQR: Interquartile range
BAU: Binding antibody units.
