# Supplementary material for: Impaired Antibody Response Following the Second Dose of the BNT162b2 Vaccine in Patients With Myeloproliferative Neoplasms Receiving Ruxolitinib
Source: Front Med (Lausanne). 2022 Mar 25;9:826537. doi: 10.3389/fmed.2022.826537 (PMC8990027; doi:10.3389/fmed.2022.826537)
Supplement: Supplementary file 1 [file Table_1.DOCX]

Supplementary Material

# Supplementary Tables

**Supplementary Table 1.** Lymphocyte subset analysis in MPN patients with or without ruxolitinib

|  | MPN-Ruxo (n=10) | MPN-no Ruxo (n=26) | *p* |  | All MPN patients (n=36) |
| --- | --- | --- | --- | --- | --- |
| WBC (/μL, IQR) | 4300 (3225-9350) | 7300 (5900-10825) | 0.08 |  | 7050 (4800-10475) |
| Lymphocyte (/μL, IQR) | 928 (854-1074) | 1318 (1095-1734) | 0.003 |  | 1138 (980-1575) |
| IgG (mg/dL, IQR) | 1253 (1046.7-1424.0) | 1411 (1102.0-1588) | 0.321 |  | 1299 (1093-1550) |
| CD19^+^ cell (/μL, IQR) | 90.3 (68.7-133.6) | 104.7 (84.6-164.0) | 0.303 |  | 104.1 (75.7-154.9) |
| CD3^+^ cell (/μL, IQR) | 613.1 (426.0-679.9) | 800.6 (676.1-1257.5) | 0.007 |  | 703.9 (589.8-1012.6) |
| CD4^+^ cell (/μL, IQR) | 290.2 (239.8-388.9) | 500.0 (394.3-786.3) | 0.007 |  | 472.5 (314.1-660.7) |
| CD8^+^ cell (/μL, IQR) | 168.2 (158.0-212.4) | 251.1 (190.7-319.9) | 0.056 |  | 231.7 (159.2-277.0) |
| CD4^+^ cell / CD8^+^ cell | 0.19 (0.13-0.24) | 0.15 (0.11-0.23) | 0.614 |  | 0.16 (0.11-0.24) |
| CD56^+^ cell (/μL, IQR) | 65.3 (48.1-129.5) | 160.8 (102.4-283.5) | 0.002 |  | 134.9 (79.5-256.3) |

MPN; myeloproliferative neoplasms, WBC; white blood cell, IgG; immunoglobulin G, IQR; interquartile range
