# Supplementary material for: Impaired Antibody Response Following the Second Dose of the BNT162b2 Vaccine in Patients With Myeloproliferative Neoplasms Receiving Ruxolitinib
Source: Front Med (Lausanne). 2022 Mar 25;9:826537. doi: 10.3389/fmed.2022.826537 (PMC8990027; doi:10.3389/fmed.2022.826537)
Supplement: Supplementary file 2 [file Table_2.DOCX]

Supplementary Material

# Supplementary Tables

**Supplementary Table 2.** Univariate analysis of lymphocyte subset for obtaining the protective level of COVID-19 antibody after 2 doses of vaccination

| MPN patients (n=36) | Univariate analysis | | |
| --- | --- | --- | --- |
|  | Odds ratio | 95%CI | *p* |
| Lymphocyte < 1100 (/μL) | 0.53 | 0.14-2.06 | 0.366 |
| CD19^+^ cell < 100 (/μL) | 0.41 | 0.10-1.59 | 0.198 |
| CD3^+^ cell < 700 (/μL) | 0.14 | 0.03-0.64 | 0.011 |
| CD4^+^ cell < 470 (/μL) | 0.07 | 0.01-0.39 | 0.001 |
| CD8^+^ cell < 230 (/μL) | 1.26 | 0.33-4.74 | 0.735 |
| CD56^+^ cell < 130 (/μL) | 0.5 | 0.13-1.92 | 0.313 |

MPN: myeloproliferative neoplasms; CI: confidence interval.
